# The Brazilian Journal of Physical Therapy is now published by Elsevier: a step forward

**DOI:** 10.1590/bjpt-rbf.2014.0196

**Published:** 2016

**Authors:** Paula Rezende Camargo, Leonardo Oliveira Pena Costa, Sergio Teixeira Fonseca

The Brazilian Journal of Physical Therapy (BJPT) overcame a large number of challenges since its creation in 1996. Today, the BJPT is indexed to all major databases and has an impact factor ranging from 0.9 to 1.0 over the last 4 years.

Thanks to the effort of all authors and members that served the Editorial Board during these 20 years, the BJPT has continuously growing in quality. Great effort has also been put to reduce the review time and to publish high quality papers that could influence clinical practice. Despite all accomplishments, it is time for another step forward, which will help improving the worldwide visibility of the articles published by the BJPT. Thus, it is our pleasure to announce the partnership between the BJPT and Elsevier, starting January 2017.

Elsevier is the largest scientific publisher worldwide. Elsevier currently publishes about 2,500 journals and all Elsevier-published articles can be accessed at Science Direct free of charge for subscribers. We are strongly confident that the exposure of BJPT will increase dramatically with this new partnership.

Elsevier will also offer a new website for a better navigation of the BJPT content for the readers. EVISE® will be the new submission platform and will allow a more friendly interaction among authors, reviewers and the Editorial Board, during the process of submission until the final acceptance of the paper. As a recognition to the volunteer work of the reviewers, they will be provided with access to Scopus and Science Direct for 30 days.

Initially, the articles published by the BJPT will only be available, by subscription, through ScienceDirect, which is considered the world's largest electronic collection of science, technology, and medicine full-text and bibliographic information. However, authors will have the choice of having their papers as *Open Access* upon payment. After a 12-month embargo, all papers will become *Open Access* and accessible to the public without charges. Finally, exposure of all BJPT papers will be enhanced, as accepted papers will be rapidly published as ahead of print.

We are excited and confident that these changes are for the better, and we hope that everyone who helped us to build the success story of the BJPT will continue contributing to the journal in this major step forward.

